# Where to look for the morals in markets?

**DOI:** 10.1007/s10683-019-09608-z

**Published:** 2019-03-19

**Authors:** Matthias Sutter, Jürgen Huber, Michael Kirchler, Matthias Stefan, Markus Walzl

**Affiliations:** 1 Max Planck Institute Bonn, University of Cologne, Cologne, Germany; 2 University of Innsbruck, Innsbruck, Austria; 3 University of Gothenburg, Gothenburg, Sweden; 4 Max Planck Institute for Research on Collective Goods, Kurt Schumacher Strasse 10, 53113 Bonn, Germany

**Keywords:** Morals, Markets, Competition, Experiment, C92, D03, D62

## Abstract

There is a heated debate on whether markets erode social responsibility and moral behavior. However, it is a challenging task to identify and measure moral behavior in markets. Based on a theoretical model, we examine in an experiment the relation between trading volume, prices and moral behavior by setting up markets that either impose a negative externality on third parties or not. We find that moral behavior reveals itself in lower trading volume in markets with a negative externality, while prices mostly depend on the market structure. We further investigate individual characteristics that explain trading behavior in markets with negative externalities.

## Introduction

In the early history of economic thought, some of the most important founders of modern economics dealt extensively with the relationship between markets and morals. Depending on the analysis, some scholars arrived at rather opposite conclusions. For instance, while Adam Smith argued that markets would, in principle, have a civilizing effect on the behavior of market participants (Smith 1763), Karl Marx and Thorsten Veblen expected markets to be destructive and bring out the worst in human beings (Marx 1867; Veblen 1899). Given the ubiquity of markets in our daily life, the question of how they affect human, and in particular moral, behavior is an immensely important one. Yet, during the second half of the twentieth century, the question of how markets relate to morals was relegated to the background of the academic debate. Only during the past decade the academic community has rediscovered this topic, probably fueled by scandals like Enron (Healy and Palepu 2003), the revelation of massive child labor as a backbone of the global textile industry (Edmonds 2007), or more recent scandals in the finance industry (Cohn et al. 2014). For instance, Shleifer (2004) has argued that the competitive pressure in markets creates strong incentives for unethical practices (like child labor, tax evasion or corruption) to reduce costs and thus guarantee survival in a competitive environment. In addition, Sandel (2012) has claimed that markets—or more generally price mechanisms—might undermine moral values per se by crowding out norms such as respect for human life and dignity.

Using experimental methods, Falk and Szech (2013) were the first to demonstrate under controlled laboratory conditions that, indeed, markets can undermine moral values. More precisely, they let subjects decide whether to take some money and let a mouse be killed or forgo money and let the mouse live. The focus of their work was on comparing behavior when subjects decided individually and when they traded on bilateral or multilateral double-auction markets. First, they found that subjects were more frequently willing to let a mouse be killed in a double auction market than when making an individual decision. Second, they reported a downward trend in prices in the multilateral markets, which they interpreted in the following way: “The downward trend provides a further indication of moral decay in the mouse market and is suggestive of social learning and endogenous social norm formation. Intuitively, observing low trading prices in the market may make it normatively acceptable to offer or accept low prices as well.*”* (Falk and Szech 2013, p. 709).^1^

In this paper, we start from their interpretation and investigate how moral behavior in markets influences aggregate market prices and quantities traded. In order to do so, we keep the general simplicity of the design of Falk and Szech (2013)—by letting buyers and sellers trade in a multilateral double auction market where trading has a negative externality—and add a treatment variation that is completely identical, except that we remove the negative externality. This creates the simplest possible environment to assess how a negative externality affects aggregate market outcomes. Given the growing literature in this field, this question is relevant from a methodological viewpoint. Furthermore, since policy interventions take place in specific market settings, understanding the intricate relation between moral behavior and market outcomes is also relevant from a practical viewpoint.

In our experiment, we let buyers and sellers trade in a double auction market in a sequence of ten periods to split a fixed sum of money between a buyer and a seller. We implement a 3 × 2-between-subject design: With the first treatment-variable we vary the number of buyers and sellers such that there are either more buyers than sellers, more sellers than buyers, or an equal number of sellers and buyers. With the second treatment-variable we vary whether concluding a trade triggers an externality or not. Thus, in half of the markets striking a deal has only the consequence of distributing money between the buyer and the seller. In the other half, a deal entails the additional negative externality of voiding donations for a potentially life-saving vaccine that is provided by UNICEF to reduce the death toll of about 90,000 people that die each year because of measles (see the statistics for 2016 in the World Health Organization’s factsheet at http://www.who.int/mediacentre/factsheets/fs286/en/).

By systematically varying the number of buyers and sellers, we investigate how the competitive pressure on each market side influences aggregate market outcomes, in particular prices and trading volume. We compare the price developments in markets with and without an externality, holding the number of buyers and sellers constant. This feature lets the number of buyers and sellers who trade in the market be endogenously determined, and it allows investigating whether the externality creates a difference in trading volume or trading prices over time. In this way, we can disentangle the impact of competitive pressure and of moral concerns on market outcomes.

As predicted by a simple model of price-taking behavior by agents with an aversion against generating a negative externality from trade, we find that the presence of an externality reduces the trading volume but that the effect on prices depends on the market structure. If there is an equal number of buyers and sellers, prices remain unaltered. In contrast, if there are more buyers than sellers, competitive pressure between buyers increases prices, but this effect is moderated in the presence of an externality. If there are more sellers than buyers, the effect is reversed.

With our study we contribute to the emerging experimental literature focusing on the interplay of morals and markets. Following the seminal work of Falk and Szech (2013), several recent studies have tried to identify why markets might erode moral values. Among the most important explanations are diffusion of responsibility and lack of pivotality in markets, social information about the acceptability of a particular (unethical) behavior, or market framing that distracts attention from the moral dimension of the traded good (Bartling et al. 2015, 2017; Breyer and Weimann 2015; Cappelen et al. 2017; Falk and Szech 2015; Gneezy et al. 2014; Irlenbusch and Saxler 2015; Irlenbusch and Villeval 2015; Kirchler et al. 2016). Although many of these studies discuss certain aspects of morals in markets, none of them did examine the interplay of market structure, moral behavior and aggregate market outcomes in detail. We contribute to this line of literature by showing that morals in markets reveal itself in lower trading volume, while prices mostly depend on the market structure. Declining prices are not a straightforward indicator of declining morals in markets of the Falk and Szech (2013) paradigm, but rather have to be reviewed in light of the relative market power of buyers and sellers (as their markets had two more sellers than buyers).

In the next section, we introduce our experimental design and the details of the moral externality as well as our hypotheses derived from a simple model. Section 3 presents the experimental results and examines trading volume and prices separately. Furthermore, in Sect. 3 we also discuss trader characteristics that are relevant for trading behavior in markets with an externality. Section 4 discusses our results and concludes the paper.

## Experimental design and hypotheses

### Treatments *without* an externality

We conduct three treatments where trading in a market does *not* generate a negative externality on an uninvolved third party. In all of these treatments, there are ten traders in the market, either in the role of buyer or seller. Each of them can place limit orders and accept them by posting market orders. These orders indicate how a fixed sum of 21.40 Euro shall be divided between a buyer and a seller. More precisely, buyers and sellers can submit orders to agree on a price *P* that has the following consequence: the seller receives *P* Euro as payment, and the buyer gets the remaining pie, i.e., 21.40—*P* Euro. Trading rules are identical to Kirchler et al. (2016)^2^ and as in a classic double auction market: orders are executed according to price and then time priority. Market orders have priority over limit orders and are always executed instantaneously. The trading screen provides real-time information about the current price and about the number of transactions in the period (see the instructions in the Online Appendix).

Each trader can conclude at most one trade per period. Once this is the case, this trader’s remaining open limit orders are removed from the order book and she cannot enter new orders. Each trading period lasts for three minutes. In total, subjects trade for ten periods. At the end of the experiment, one period is drawn randomly and implemented with all monetary consequences. If a subject has not traded in the randomly drawn period, then her earnings are zero. The three treatments differ with respect to the number of buyers and sellers in the market.

- SYMM has five sellers and five buyers, implying a maximum of five trades per period.
- 6SELLERS has six sellers and four buyers, allowing for a maximum of four trades per period.
- 6BUYERS has four sellers and six buyers, entailing four trades per period at most.

### Treatments *with* an externality

The three treatments *with* an externality also have ten traders each. Buyers and sellers can submit prices, and if a pair of them concludes a trade, the pie of 21.40 Euro is split according to price *P*. However, whenever a trade occurs, this triggers the externality that there will be no donation of 21.40 Euro to UNICEF for financing one package with 100 doses of (potentially life-saving) measles vaccine. One such package is sufficient to vaccinate 50 children twice, which yields full protection against measles. Thus, traders in these treatments face a trade-off between a monetary payment if a trade is concluded and avoiding a negative moral externality if no trade occurs. In Kirchler et al. (2016) we reported questionnaire evidence showing that in markets with an externality trading is considered as significantly less moral than not trading. Thus, moral norms are not imposed by us, but are rather shared by the majority of experimental subjects.^3^

The three treatments with the externality are analogous to those without the externality.

- SYMM_EXT has five sellers and five buyers.
- 6SELLERS_EXT has six sellers and four buyers.
- 6BUYERS_EXT has four sellers and six buyers.

### Model and hypotheses

To derive testable hypotheses, we analyze a simple model of price-taking behavior in a double auction market. Consider a market with *m* > 0 sellers and *n* > 0 buyers. Buyers have unit demand and sellers have unit supply of a homogenous good. Each buyer’s valuation of the good is *v* (= 21.40 EUR in our experiment), each seller’s cost is 0. Buyers and sellers may differ in the extent to which they (1) dislike an unequal distribution of the surplus from trade (see Franciosi et al. 1995; Borck et al. 2002; Cason et al. 2011) and (2) internalize the externalities they generate by trading with each other. Suppose each buyer and seller has a type *t* distributed with full support on [0, 1] according to a cumulative distribution function *G*(*t*). Without an externality from trade, a buyer’s willingness to pay for the transaction is set to be *v* − *tg* with *v*/2 > *g* > 0. I.e., a buyer of type *t* faces trading costs *tg* such that she/he prefers not to trade rather than leaving more than *v* − *tg* of the surplus to the seller. The larger *g*, the fewer types are willing to pay high prices (and correspondingly are willing to accept a small fraction of the surplus from trade). This captures fairness considerations (Fehr and Schmidt 1999; Bolton and Ockenfels 2000; Charness and Rabin 2002; Cooper and Kagel 2016) in a tractable way. Likewise, a seller’s cost (or willingness to accept) is *tg*, indicating that a seller of type *t* prefers not to trade rather than leaving more than *v* − *tg* of the surplus to the buyer. If trade induces an externality *h* > 0, the willingness to pay of a buyer of type *t* becomes *v* − *t*(*g* + *h*) and the willingness to accept of a seller of type *t* becomes *t*(*g* + *h*), respectively.

If the market is competitive, i.e., buyers and sellers are price-takers and trade occurs at a market-clearing price, buyers offer their willingness to pay and sellers ask for their costs. As types *t* are drawn from *G*(*t*), this generates a downward sloping demand and an upward sloping supply. If there is no externality (i.e., *h* = 0), demand and supply do not intersect because a buyer’s willingness to pay is (for all *t* < 1) larger than *v*/2 and a seller's willingness to let is (for all *t* < 1) smaller than *v*/2. As a result, the trading volume is maximal, i.e., *min*(*m*,*n*) (for a proof see Result 1 in Appendix 1).

#### **H1**

For h = 0, i.e., when there is no externality in the market, trading volume is at its maximum, i.e., 4 for the asymmetric treatments and 5 for SYMM.

Introducing an externality (i.e., *h* > 0) reduces the willingness to pay of a buyer with type *t* from *v* − *tg* to *v* − *t*(*g* + *h*) and enhances the costs of a seller of type *t* from *tg* to *t*(*g* + *h*). As *h* is increasing, demand and supply become steeper and intersect at a smaller trading volume (for a proof see Result 2 in Appendix 1).

#### **H2**

Trading volume is lower in treatments with externality compared to the corresponding treatments with the same buyer/seller ratio and without externality.

With respect to prices, the symmetry of the market for *m* = *n* implies that the expected market clearing price (if all market clearing prices are equally likely for a given profile of types) is *v*/2 independent of the externality (see Result 3 in “Theoretical framework” of Appendix). As usual and for any given level of the externality *h*, expected market clearing prices are larger than v/2 if the number of buyers exceeds the number of sellers and expected market clearing prices are smaller if the number of sellers exceeds the number of buyers (see Result 4 in Appendix 1). In our model with price-taking buyers and sellers this is only driven by the fact that the average willingness to pay of a buyer who actually trades is larger (smaller) than the average cost of a seller who actually trades if the buyers are at the long (short) market side. If traders (gradually) learn to act strategically, we expect short market side traders to manipulate prices in their favor leading to increasing prices when there are more buyers than sellers and decreasing prices when there are more sellers than buyers. Compared to the situation without an externality (i.e., *h *= *0*), introducing an externality reduces the expected market clearing price if there are more buyers than sellers and enhances the expected market clearing price if there are more sellers than buyers whenever the externality is not too pronounced (in the model it is sufficient that* h* < (*v* − 2 *g*)—for a proof see Result 5 in Appendix 1). We summarize these findings in the following Hypothesis.

#### **H3**

Market prices in treatments SYMM_EXT and SYMM are identical and not different from the fair split, i.e., 10.7; prices in 6BUYERS_EXT exceed 10.7 and are lower than in 6BUYERS; prices in 6SELLERS_EXT are below 10.7 and are higher than in 6SELLERS.

This framework is extendable to a setting where types of buyers and sellers are drawn from different distributions to account for the possibility that traders’ aversion against generating an externality may have different origins (e.g., buyers may be intrinsically motivated while sellers could be mainly concerned about their reputation). Hypotheses 1 and 2 regarding the trading volume are unaltered by this assumption (see Results 1 and 2 in Appendix 1). With respect to Hypothesis 3, it can be shown that prices are larger in 6BUYERS than in SYMM and prices are larger in SYMM than in 6SELLERS (for any level of the externality—see Result 4 in Appendix: Theoretical Framework). Furthermore, prices in 6BUYERS (6SELLERS) decrease (increase) in the externality as long as traders care about the externality at all and the externality is not too pronounced (see Result 5 in Appendix 1). Only the finding that prices remain at the equal split in all SYMM treatments regardless of the externality crucially relies on the assumption that types are drawn from identical distributions. If, for instance, (almost) all sellers have types close to zero, expected prices in SYMM treatments would be below the equal split and decreasing in the externality.

### Side experiments

In addition to the market experiment, we ran the following three side experiments to gather data on individuals’ characteristics that potentially can explain their market behavior:

First, we measured risk-attitudes in a standard choice-list setting (Bruhin et al. 2010; Dohmen et al. 2011). Subjects could choose between a risky alternative, yielding either zero or 6 Euro with equal probability, and a safe payment that increased from 0.5 Euro to 6 Euro in steps of 0.5 Euro. The more risk averse an agent is, the more likely it is that the agent does not trade.

Second, we measured subjects’ willingness to compete, following the seminal design of Niederle and Vesterlund (2007) and implementing the parameters of Balafoutas and Sutter (2012). There were three stages, with feedback given only at the very end. In a first stage, subjects had to add up sets of five double-digit numbers within 2 min, and were paid 0.5 Euro for each correct solution. In a second stage, they had to compete in pairs of two, with only the winner getting paid 1 Euro per correct solution. In a third stage, subjects could choose whether they wanted to be paid a piece rate as in stage 1 or according to the competitive scheme in stage 2. The latter choice is interpreted as a subject’s willingness to compete, and this trait might be related to behavior in our experimental markets.

Third, we ran a dictator game where subjects had to decide how to split 5 Euro between themselves and another, anonymous participant in the room. Only after having taken the decision, their role in the dictator game as either dictator or recipient was revealed, i.e., we applied the strategy method (Brandts and Charness 2011). The dictator game was used to elicit distributional preferences because they might influence whether and how a subject wants to split the fixed sum of 21.40 Euro in the market treatments.

At the end of a session, one of the three side experiments was selected randomly for payment. If the risk experiment was chosen, it was also determined which choice was relevant (one out of twelve choices). If the experiment on the willingness to compete was chosen, it was also randomly determined which stage was payoff-relevant.^4^

### Experimental procedure

For each of our two experimental treatments with an equal number of buyers and sellers (SYMM and SYMM_EXT) we conducted eight markets with ten subjects each and for the four treatments with an unequal number of buyers and sellers we had 12 markets with ten subjects each. No subject was allowed to participate in more than one session, i.e., we used a between-subject design. In total, 640 bachelor and master students from various fields of study participated in the experiment, using ORSEE by Greiner (2015) and HROOT by Bock et al. (2014) for recruitment. All sessions were run at the Innsbruck EconLab at the University of Innsbruck using zTree (Fischbacher 2007).

Each experimental session lasted between 60 and 90 min. At the beginning, subjects had 15 min to read the instructions on their own and questions were answered privately. Afterwards, the trading screen was explained, followed by a non-incentivized trial period of 3 min to become familiar with the trading interface. After subjects had read the instructions, they had the possibility to leave the experiment if they did not want to participate (only in the treatments with an externality). Subjects who left the experiment received the show-up fee of 10 Euro and were replaced with reserve candidates. The latter were assigned the roles of reserves before the experiment started, but were present from the beginning (i.e. they also read the instructions and had the same information as all other experimental subjects). In sum, only nine out of 320 participants in the treatments with an externality left a session and were replaced by reserve candidates.^5^ No reference to “morals” or any other similar term was used in the experiment (see the instructions in the Online Appendix for details).

In addition and subsequent to the market experiment, we ran the three side experiments and administered a questionnaire at the end of each experimental session to control for various economic preferences and background information.

At the end of the experiment subjects had to answer a questionnaire about background variables (see Appendix 1). In addition to a show-up fee of 10 Euro, subjects received the payments from the market experiment and from one randomly drawn side experiment in private and anonymously by a researcher who was not in the room during the experiment. The average total payment was 21.72 Euro per subject.

In the treatments with an externality, subjects were informed in the instructions that we would send them a receipt about the amount donated in the sessions within the next 2 months. In total, we donated 920.20 Euro to UNICEF, making 4300 measles vaccinations possible, thus protecting 2150 children from a measles infection.

## Results of the experiment

### Trading volume

**Fig. 1:**
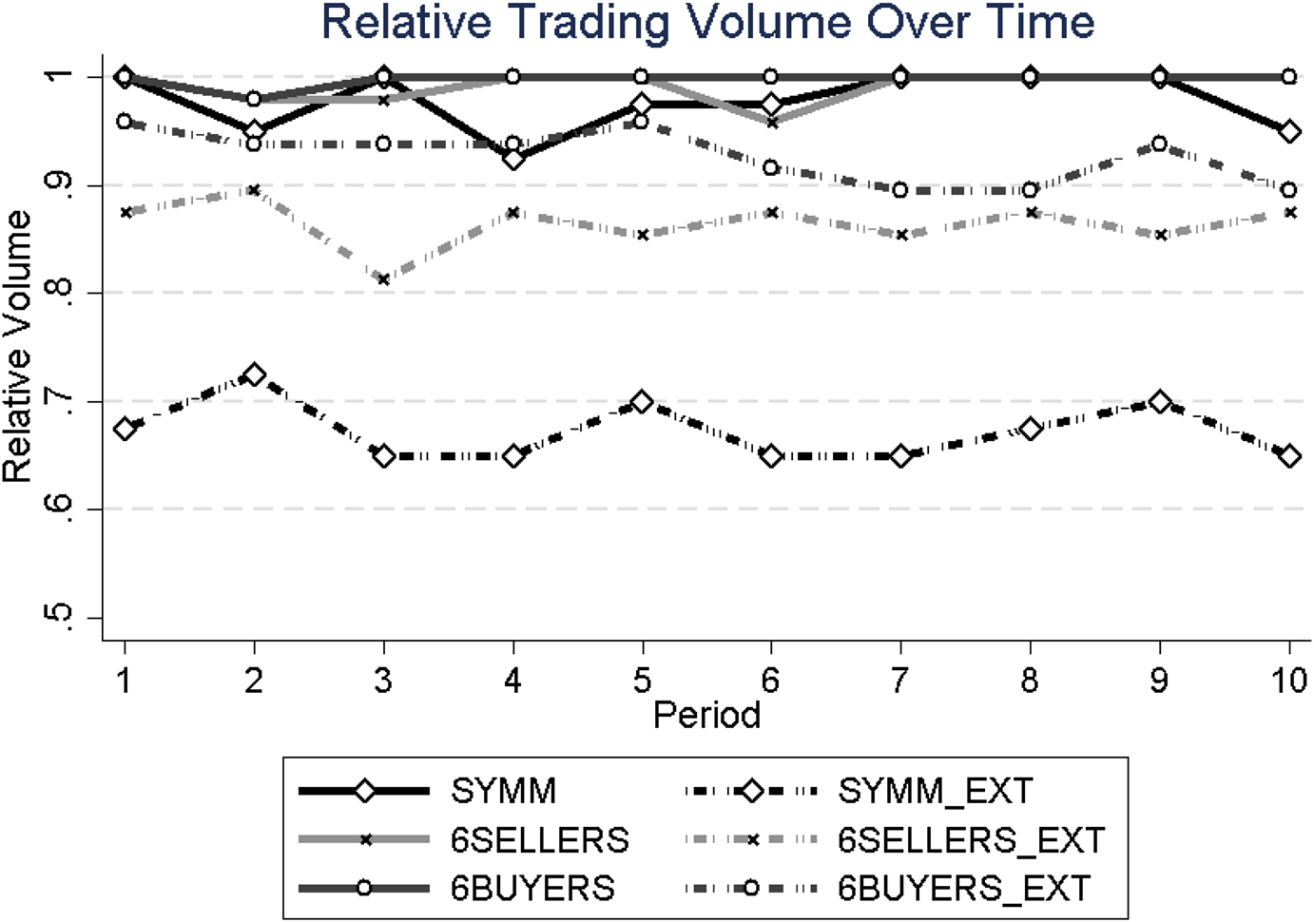
Average relative trading volume across periods

Figure 1 presents the average relative trading volume per period, calculated as the actual number of trades divided by the maximum number of trades possible, which is four in the treatments with an unequal number of sellers and buyers, and five in the symmetric treatments. While—over all periods—all treatments *without* an externality have mean relative trading volumes close to 100% (ranging from 97.75% in SYMM to 99.79% in 6BUYERS), i.e., corroborating hypothesis H1, the treatments *with* an externality have considerably lower relative trading volumes, ranging from 67.25% in SYMM_EXT to 92.71% in 6BUYERS_EXT. Using the average relative trading volume across the ten periods of each market as the unit of observation and testing for pairwise differences in the trading volume with Mann–Whitney *U* tests, we find significantly lower trading volumes in the treatments *with* the externality (*p* < 0.001 for SYMM vs. SYMM_EXT, *N* = 16; *p* = 0.018 for 6SELLERS vs. 6SELLERS_EXT, *N* = 24; *p* = 0.021 for 6BUYERS vs. 6BUYERS_EXT, *N* = 24). Table 1 confirms these non-parametric results. It presents three fraction (logit) panel regressions (see Papke and Wooldridge 1996), with clustered standard errors on the market level, separately for the three sets of matched treatments (with and without externality), and with the relative trading volume as dependent variable. As explanatory variables we include a dummy for whether the market has an externality (EXT), PERIOD for periods 1 to 10, and an interaction term of PERIOD and EXT to account for potentially different trading volume developments in treatments with and without externality. We find that the relative trading volume is significantly lower when an externality arises from trading (see the significant dummy EXT in the first two columns and the significant negative interaction term in the third column), which can also be seen in Fig. 1.

**Table 1:** Regressions on relative trading volume

	(1) SYMM and SYMM_EXT	(2) 6SELLERS and 6SELLERS_EXT	(3) 6BUYERS and 6BUYERS_EXT
EXT (= 1)	− 2.82 (0.37)***	− 2.14 (1.07)**	− 0.83 (1.19)
PERIOD	0.03 (0.08)	0.16 (0.09)*	0.69 (0.00)***
PERIOD*EXT	− 0.04 (0.09)	− 0.16 (0.09)*	− 0.78 (0.04)***
Constant	3.59 (0.33)***	4.00 (1.00)***	3.87 (0.99)***
*N*	160	240	240

Fraction (logit) regression with clustered standard errors on market level. Dependent variable is the relative trading volume. The total number of trades in a period is divided by the maximum number of trades (5 in SYMM and SYMM_EXT, and 4 in the other treatments), thus ranging from 0 to 1. Coefficients are reported. Robust standard errors are given in parenthesis

*,**,*** represent the 10%, 5% and the 1% significance levels

Taken together, Fig. 1 and Table 1 show a clear effect of the externality on trading volume as indicated by hypothesis 2.

From Fig. 1, one can see that the relative trading volume is clearly lower in SYMM_EXT than in either 6SELLERS_EXT or 6BUYERS_EXT (*p* < 0.02 in each pairwise comparison; Mann–Whitney *U* tests; *N* = 20), whereas the relative trading volume between 6SELLERS_EXT and 6BUYERS_EXT is not significantly different (*p* = 0.378; Mann–Whitney *U* test; *N* = 24). Hence, trading volume drops more strongly with an externality when the number of sellers and buyers is equal than when their numbers are unequal. The larger reduction in trading volume in SYMM_EXT compared to the asymmetric treatments (6BUYERS_EXT and 6SELLERS_EXT) may be due to the higher pivotality of traders in the former. If one of the ten traders refuses to trade (e.g., for moral reasons), this implies a reduction in trading volume by 20% (one out of five possible trades) in SYMM_EXT. In the asymmetric treatments, there is only a reduction if the trader is on the shorter side.^6^ In this case there is a reduction by 25% (one out of four trades), but if she is on the longer side there is no reduction of trading volume at all. As the chance to be on the shorter side is 40% (four out of ten traders), in the asymmetric treatments trading volume falls, on average, by only 10% (25% times 0.4) if one trader refuses to trade, compared to 20% reduction in SYMM. This may explain why the trading volume drops more strongly in case of an equal number of buyers and sellers.

In line with the results on trading volume, the fraction of subjects who rarely or never trade differs widely between the treatments with and without externality. In treatments with externalities, 10.63% of subjects never trade, and a further 5.31% trade only once or twice (out of ten periods). In comparison to these 15.94% of subjects with at most two trades, there are only 1.88% of subjects with two or less trades in the treatments without an externality. Overall, each subject trades on average in 7.02 periods when there is an externality, but in 8.56 periods when there is none,^7^ and this difference is highly significant (*p* < 0.001, Mann–Whitney *U* test with the average relative trading volume in each market as unit of observation, *N* = 64). These differences in the individual willingness to trade generate the lower trading volume in the treatments with an externality. Thus, we find evidence supporting our hypothesis H2: in markets with an externality, overall trading volume drops and the number of subjects refusing to trade increases significantly. Since the monetary incentives for traders are the same in both sets of treatments, the reduction of trade in treatments with an externality can be interpreted as an indication of moral behavior. The next question is how the introduction of moral externalities influences market prices.

### Market prices

**Fig. 2:**
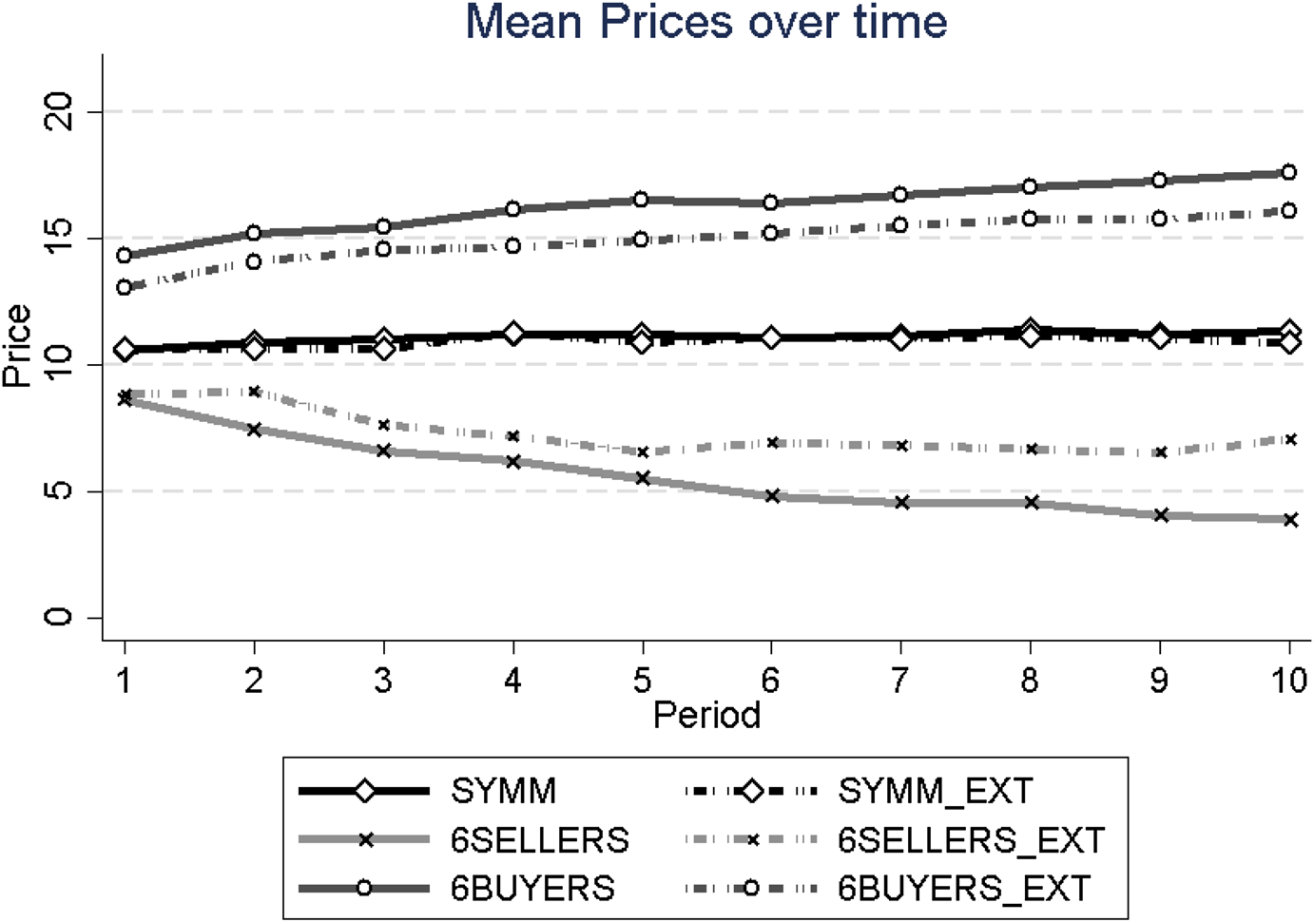
Mean prices across periods

Figure 2 presents the average transaction prices per period in each of the six treatments. The first finding to notice is that average prices are lowest when there are more sellers than buyers (in 6SELLERS and 6SELLERS_EXT), intermediate when the number of buyers and sellers is equal (in SYMM and SYMM_EXT), and highest when there are more buyers than sellers (in 6BUYERS and 6BUYERS_EXT).^8^ This ordering of prices is as expected in hypothesis H3, and the differences are all significant.^9^

In Fig. 2, one can see that average prices are very close in each pair of corresponding treatments. This is rather different from the findings with respect to trading volume, where the externality led to significantly lower relative volume. No pairwise comparison of average prices is significant (*p* = 0.75 for SYMM vs. SYMM_EXT, *N* = 16; *p* = 0.11 for 6SELLERS vs. 6SELLERS_EXT, *N* = 24; and *p* = 0.12 for 6BUYERS vs. 6BUYERS_EXT, *N* = 24; Mann–Whitney *U* tests).

Figure 2 also reveals price dynamics, i.e., the development of mean prices over the ten periods, which have been argued by Falk and Szech (2013) to be an indicator of decreasing  morals. Here we see that prices are already different between treatments in the first round—as a consequence of the different level of competitive pressure on a particular market side—and then drift apart steadily, with prices in the symmetric treatments staying essentially constant across all periods. As expected from the discussion in Sect. 2.3 (and experiments without externality by, e.g., Cason and Williams 1991), prices in markets with more buyers than sellers increase over time, while those with more sellers than buyers decrease over time.

Table 2 presents a GLS panel regression with clustered standard errors on the market level, separately for three paired treatments (with and without externality). The dependent variable is the mean market price per period. As explanatory variables we include a dummy for whether the market has an externality (EXT), PERIOD for periods 1 to 10, and an interaction term of PERIOD and EXT to account for potentially different price developments in treatments with an externality. Column (1) refers to the treatments with an equal number of sellers and buyers. Here we note that EXT is insignificant and so is the interaction term PERIOD*EXT. PERIOD is significant, but of small magnitude (coefficient: 0.07), reflecting the slight increase of average prices from 10.6 in period 1 to 11.2 in period 10.

**Table 2:** Regressions on mean prices

	(1) SYMM and SYMM_EXT	(2) 6SELLERS and 6SELLERS_EXT	(3) 6BUYERS and 6BUYERS_EXT
EXT (= 1)	− 0.06 (0.84)	0.23 (0.83)	− 1.10 (0.71)
PERIOD	0.07 (0.03)**	− 0.50 (0.04)***	0.33 (0.05)***
PERIOD*EXT	− 0.02 (0.11)	0.26 (0.10)***	− 0.04 (0.10)
Constant	10.76 (0.62)***	8.37 (0.60)***	14.48 (0.55)***
*N*	160	240	240

GLS panel regressions with clustered standard errors on market level. Dependent variable is the mean price in each period. Coefficients are reported. Robust standard errors are given in parenthesis

*,**,*** represent the 10%, 5% and the 1% significance levels

Column (2) refers to the two treatments with six sellers. Again, EXT is insignificant, while the PERIOD-variable is significantly negative, as prices decrease over the course of the experiment. Here, the interaction term is also significant, and positive, since the decline in prices is less marked (and prices therefore closer to the equal split) when externalities arise from trading.^10^

Column (3) refers to the treatments with six buyers, and here we only see a significant PERIOD-variable, showing that prices increase over the ten periods, but neither EXT nor the interaction term are significant. Overall, we find mixed support for our hypothesis H3: Prices do not significantly differ between SYMM and SYMM_EXT. Price trends are less marked in 6BUYERS_EXT and 6SELLERS_EXT compared to 6BUYERS and 6SELLERS, respectively, but only significantly so for 6SELLERS_EXT. Note, however, that in Appendix 1 we demonstrate that a significant difference between prices in 6BUYERS and 6BUYERS_EXT or 6SELLERS and 6SELLERS_EXT can only be expected if the externality is not too large. In that sense, the insignificant impact of the externality on prices when buyers are the long market side is, nevertheless, in-line with our simple model.

Recall that Falk and Szech (2013) argued that the falling prices observed in their mouse market are an indicator of decreasing morals. Note that in their markets there were two more sellers than buyers, as in our 6SELLERS_EXT treatment. Our data suggest that the mere fact of falling prices is not a good and unambiguous indicator for decreasing morals for two reasons. First, there is already a price decline when there is no externality in 6SELLERS, a treatment that differs from 6SELLERS_EXT only in that it has no externality from trading. Second, when there are more buyers than sellers in the market, prices increase even with an externality. Hence, we prefer to interpret the price dynamics (falling or increasing) as the expected outcome when the competitive pressure on each market side changes with the number of buyers and sellers, but that price dynamics are not indicative of increasing or decreasing morals. This is all the more evident when we combine the price dynamics with the development of relative trading volume. Recall first that prices are falling in 6SELLERS_EXT and increasing in 6BUYERS_EXT. These price dynamics imply that trading becomes more and more attractive (in monetary terms) for the shorter market side of four buyers in 6SELLERS_EXT, respectively of four sellers in 6BUYERS_EXT, because traders on the shorter market side make higher profits when prices become more extreme (i.e., lower in 6SELLERS_EXT and higher in 6BUYERS_EXT). If this is the case, the relative trading volume should *increase* as a consequence of the observed price dynamics, because at the margin traders on the shorter market side should be even more compensated for the moral costs and, thus, more likely willing to trade rather than abstain from trading. Yet, Fig. 1 and Table 1 show that this is not the case. If anything, the relative trading volume with an externality is *decreasing* across periods, despite the more attractive prices and the increasing compensation for moral costs for the shorter market side. We believe that this shows that price dynamics are not a straightforward indicator of decreasing moral values.

### Impact of those who refuse to trade

Column (1) of Table 3 shows GLS panel regression of trading volume over time and Panel 2 shows similar regression with prices over time as dependent variable.^11^ The explanatory variables indicate the number of subjects refusing to trade in any period on the short and long side of the market, REFUSE_LONG and REFUSE_SHORT, respectively. In order to include both, 6BUYERS_EXT and 6SELLERS_EXT, we normalize prices as gains for the shorter market side (i.e.,* p* in case of 6BUYERS_EXT and *21.4-p* in case of 6SELLERS_EXT).

**Table 3:** Regressions refusers of trading

	(1) Trading volume	(2) Prices (normalized)
REFUSE_SHORT	− 1.13 (0.12)***	1. 30 (1.10)
REFUSE_LONG	− 0.10 (0.08)	− 1.58 (0.60)***
PERIOD	− 0.01 (0.01)	0.26 (0.06)***
Constant	3.96 (0.06)***	14.01 (0.60)***
*N*	240	240

GLS panel regressions with clustered standard errors on market level. Dependent variables are trading volume and mean price in each period, respectively. Coefficients are reported. Robust standard errors are given in parenthesis

*,**,*** represent the 10%, 5% and the 1% significance levels

One can see that subjects refusing to trade have a high and significant impact on trading volume when they are on the shorter market side. However, this pattern is reversed when it comes to prices: the impact on prices comes from subjects refusing to trade on the longer market side. The negative sign indicates that they can drive prices closer to the equal split, i.e., to “fairer” market prices.

### Individual trading behavior

So far, we have concentrated on aggregate market data. Given that we base our argumentation on subjective moral costs, it is interesting to explore the potential factors that can explain an individual’s propensity to trade in the markets with an externality. In the following analysis of individual behavior, we disregard the markets without externalities, because there trading volume is almost at 100% and because we are interested to understand which personal characteristics and economic preferences of a particular subject might be able to explain how often (out of a maximum of ten potential trades) a subject concluded a trade.

Table 4 presents results from an ordered probit regression with clustered standard errors on the market level.^12^ The dependent variable is a subject’s total number of concluded trades in the experiment, ranging from 0 to 10. As independent variables, we include gender (FEMALE = 1), field of study (taking natural sciences as the benchmark), behavior in the three side experiments and three questions from the questionnaire. Concerning the side experiments, TRANSFER measures the share of the endowment in the dictator game that is transferred to the recipient, ranging from zero to five. The relation between social behavior in the dictator game and the moral trading behavior in the markets is not straightforward: we expect prosocial behavior in both tasks to correlate, since norm-driven subjects might behave similarly in both tasks. However, it is also possible that subjects are more prosocial in the dictator game because of a bad conscience after trading in the market (Gneezy et al. 2014). RISK measures a subject’s risk preferences. This variable is calculated as the number of lotteries that are preferred over a safe amount (thus ranging from zero to twelve). We expect more risk-averse subjects to be less active in the market, since trading involves some risk. Finally, the dummy COMPETITIVE takes on the value of 1 if a subject preferred the competitive payment scheme over the piece rate in the competition experiment, and 0 otherwise. We conjecture that competitive subjects trade more in the market in order to perform better than the others, maybe even disregarding the negative externality (Charness et al. 2014). The three questions from the post-experimental questionnaire are captured by DISPOS-INCOME, NO-DONATIONS and RIGHT-WING. DISPOS-INCOME reports the disposable monthly income of subjects and is ordered in five categories from 1 to 5 (< 400, 400–800, 800–1200, 1200–1600, > 1600 Euro). A value of 1 for NO-DONATIONS indicates that a subject stated in the questionnaire that he or she had not donated in the past or does not want to donate any money to a charity. RIGHT-WING is a variable ranging from 0 for self-reported very left-wing political attitudes to 5 for very right-wing attitudes.

**Table 4:** Regressions on subjects’ number of trades

FEMALE (= 1)	− 0.08 (0.11)
TRANSFER	− 0.02 (0.04)
RISK	− 0.05 (0.04)
COMPETITIVE	− 0.12 (0.14)
DISPOS-INCOME	0.02 (0.13)
NO-DONATIONS	0.52 (0.20)***
RIGHT-WING	0.15 (0.06)**
Study law	0.19 (0.33)
Study economics and business	0.06 (0.14)
Study social sciences	0.26 (0.27)
Study medicine	0.06 (0.21)
Study humanities	− 0.20 (0.18)
*N*	300

Ordered probit regression with clustered standard errors in market level. Dependent variable is the total number of trades (ranging from 0 to 10) for each subject. Natural sciences serves as baseline study. Coefficients are reported. Robust standard errors are given in parenthesis

*,**,*** represent the 10%, 5% and the 1% significance levels

The regression results show that neither gender, field of study, disposable income, nor any of the economic preferences captured in our side-experiments have any significant explanatory power. Only two questions from the questionnaire are significant. NO_DONATIONS is significantly positive, showing that subjects who are averse to donations (by never having donated in the past or by objecting to donations in general) conclude significantly more trades than subjects who favor donations (see List 2011, for a review of the determinants of charitable giving). This is a reasonable result, since in the experiment the externality was a donation to UNICEF, which is typically regarded as a charitable organization.

The second significant variable is the (self-attributed) political attitude of a subject, measured with the variable RIGHT-WING. Subjects who consider themselves more right-wing oriented in their political attitude are more likely to conclude more trades in the course of the ten periods, and are thus more likely to trigger a negative externality, a finding reminiscent of recent work by Cappelen et al. (2017).

## Discussion and conclusion

Given the ubiquity of markets in our daily life, it is important to understand how markets affect human behavior. While markets do so in many ways, for instance by shaping the way in which we bid for objects, depending on the institutional rules of the market (Roth and Ockenfels 2002), or by influencing the level of cooperative behavior in response to exposure to market economies (Ockenfels and Weimann 1999), a powerful recent debate has revolved around the question whether markets reduce moral behavior. The main thrust of the debate seems to be the claim that markets may undermine moral behavior.

Here we have developed an experimental design that allows us to address the question how morals influence aggregate market outcomes. It has been argued that falling prices in markets for the life of a mouse indicate a decay in morals (Falk and Szech 2013). Building on their market paradigm, we have designed our experiment to provide a clean comparison of trading volume and trading prices in markets with a moral externality and other markets without such an externality in a 3 × 2 design. First, we kept the total number of traders in the market constant, but changed the ratio of buyers and sellers systematically, thus creating different levels of competitive pressure on any of the two market sides. Second, we created two sets of markets that were identical, except that one type had a negative externality if a trade was concluded, while the other one had not.

We find support for our hypothesis that moral externalities exert moral costs on experimental subjects, which in turn decreases trading volume. The effect on prices, on the other hand, depends on the market structure. The price dynamics, i.e., the decline and increase, respectively, are not a clear indicator of morals in markets. When there are more buyers than sellers, sellers gain higher profits as prices increase over time, and buyers earn more money when there are more sellers than buyers, *irrespective* of the presence or absence of a negative moral externality.

Several points might be important to note: First, in markets for fair trade products (Moore 2004), often higher prices prevail, since the production under fair and ethical conditions increases production costs (Bartling et al. 2015). For this reason, it can be argued that subjects paying higher prices reveal their valuation for the moral goods. However, the different production costs in fair trade markets justify different (typically higher) prices. In our setting, by contrast, we have kept the production costs identical in both types of markets—with and without an externality—which allows isolating the effect of moral costs on prices (and abstracting from the effect of potentially different production costs).

Second, in our analysis we assume that moral costs affect buyers and sellers equally. Although in reality it is not clear whether the rise in fair and socially responsible production is demand-driven or part of a growing social consciousness of sellers, markets might exist where moral costs are fully borne by only one side of the market. While our theoretical analysis could be expanded to such a case, we do not provide experimental evidence for it. The reason is that such a case is most exceptional, since the moral costs assumed in our analysis can also be interpreted as costs imposed by buyers on sellers, such as reputation costs.

Since it is important to gain a deeper understanding of the intricate relation between morals and markets, we consider further inquiries into this matter to be an important agenda for future research. Only with a profound understanding of this relation and with objective and reliable data, is it possible to design and implement informed policies that tackle market activities that are considered being immoral. As we find evidence that the impact of morals on prices and trading volume depends on the market structure, it seems crucial for informed policy recommendations to get a detailed understanding of the particular structure of the relevant markets, such as costs, type of markets and market power of buyers and sellers.

## Appendix: Where to look for the morals in markets?

### Appendix 1: Theoretical framework

Consider the model of price-taking behavior in a double auction market set-up in Sect. 2.3. Denote the *i*th-highest willingness to pay by *b*_*i*_ and the *j*th-lowest willingness to sell by *s*_*j*_, i.e., a profile of valuations and costs reads *b*_1_ > *b*_2_ > *…*>*b*_*n*_ and *s*_1_ < *s*_2_ < ··· <*s*_*m*_. If *k* is the largest index with *b*_*k*_ > *s*_*k*_, *k* units are traded between buyers 1,…,*k* and sellers 1,…,*k* at a (market-clearing) price in [max(*s*_*k*_, *b*_*k*+1_),min(*b*_*k*_, *s*_*k*+1_)]. Let us suppose for simplicity that any market-clearing price is equally likely.

- *Result 1: Let h* = 0. *Then*, *trading volume is min*(*m,n*).

#### *Proof*

For *h* = 0, *b*_*i*_ > *s*_*j*_ for any pair of buyer *i* and seller *j* (i.e., demand and supply do not intersect for *t* < 1) because *g* ≤ *v*/2. Therefore, the largest index *k* for which *b*_*k*_ > *s*_*k*_ is *k* = *m* for *m* ≤ *n* and *k* = *n* for *m* > *n* such that *min*(*m*,*n*) units are traded.□

- *Result 2: For any m*, *n*, *the trading volume is decreasing in h*.

#### *Proof*

For fixed *m*, *n*, and *g* ≤ *v*/2, the trading volume equals the largest index *k* for which b_k_ > s_k_. As, for all *k*, *b*_*k*_ is decreasing in *h* and *s*_*k*_ is increasing in *h*, *k* is decreasing in *h*.□

- *Result 3: Let m* = *n*. *Then*, *for any h* ≥ 0, *the expected market-clearing price is v*/2.

#### *Proof*

As all types are drawn independently from *G*(*t*) and all market-clearing prices are equally likely, this follows directly from symmetry.□

- *Result 4: For any h* ≥ 0 and *g* ≤ *v*/2, *expected market-clearing prices for m* < *n* (*n* < *m*) *are higher (lower) than expected market clearing prices for m* = *n*.

#### *Proof*

Let *m* < *n* and consider a market with *m* sellers and *m* buyers and suppose that buyer and seller types are such that *k* units are traded. Now add a buyer of type *t* [i.e. the buyer’s willingness to pay is *b* = *v*−*t*(*g *+ *h*). Prices and trading volume change depending on the relation between buyer and seller types as follows:□

#### **Case 1**

*b* < *b*_*k*+1_: Adding the buyer neither changes trade volume nor the market clearing price.

#### **Case 2**

*b*_*k*_ > *b*>*b*_*k*+1_ (we adopt the convention that *b*_*j*_ = 0 if *j* > *n*): If *b* < *s*_*k*+1_, *k* units are traded but the lower bound for market clearing prices increases from *max*(*s*_*k*_,*b*_*k*+1_) to *max*(*s*_*k*_,*b*). If *b* > *s*_*k*+1_, *k* + 1 units are traded and since *b*_*k*_ > *b*, the upper bound for a market clearing price before adding the buyer was *min*(*b*_*k*_,*s*_*k*+1_) = *s*_*k*+1_ which is a lower bound after adding the buyer.

#### **Case 3**

*b* > *b*_*k*_: If *b*_*k*_ < *s*_*k*+1_, *k* units are traded but the lower bound for market clearing prices increases from *max*(*s*_*k*_*,b*_*k*+1_) to *max*(*s*_*k*_*,b*_*k*_) = *b*_*k*_ and the upper bound increases from *b*_*k*_ = *min*(*b*_*k*_*,s*_*k*+1_) to *min(b*′*,s*_*k*+1_) *with b*′ > *b*_*k*_. If *b*_*k*_ > *s*_*k*+1_, *k* + 1 units are traded and the lower bound to market clearing prices increases from *max*(*s*_*k*_*,b*_*k*+1_) to *max*(*s*_*k*+1_*,b*_*k*+1_) while the upper bound increases from *min*(*b*_*k*_*,s*_*k*+1_) to *min(b*_*k*_*,s*_*k*+2_).

As a result, adding buyers *enhances* expected market-clearing prices. Analogously, adding a seller reduces expected market-clearing prices. In contrast, adding pairs of buyers and sellers leaves the expected market clearing price at *v*/2 (see Result 3).

- *Result 5: For* g ≤ *v*/2 *and m* < *n* (*n* < *m*), *expected market clearing prices are higher (lower) for h* = 0 *than for* 0 < *h* ≤ *v* − 2*g*.

#### *Proof*

Let *n* > *m*. For *g* ≤ *v*/2 and *h* = 0, market clearing prices are in the interval [*b*_*m*+1_,*b*_*m*_] with $b_{m} = v - t\left( {b_{m} } \right)g,\;b_{m + 1} = v - t\left( {b_{m + 1} } \right)g$, and $b_{m + 1} > v - g$. Now suppose that a price $p > v - g$ is market clearing for *h* in $\left( {0,v - 2g} \right]$. As $h \le v - 2g,\;p > h + g$. As *h* + *g* is the maximum cost for a seller (i.e., the cost of a seller of type *t* = 1), *p* > *h*+*g* implies that all *m* sellers trade and market clearing prices are in the interval $\left[ {max\left( {b_{m + 1} ,s_{m} } \right),b_{m} } \right]$. For $max\left( {b_{m + 1} ,s_{m} } \right) = b_{m + 1}$, market clearing prices are in the interval [*b*_*m*+1_,*b*_*m*_] with $b_{m} = v - t\left( {b_{m} } \right)\left( {g + h} \right) < \, v - t\left( {b_{m} } \right)g$ and $b_{m + 1} = v - t\left( {b_{m + 1} } \right)\left( {g + h} \right) < \, v - t\left( {b_{m + 1} } \right)g$. Hence, expected market clearing prices are lower than for *h* = 0 in this case. For $max\left( {b_{m + 1} ,s_{m} } \right) = s_{m}$, market clearing prices are in the interval [*s*_*m*_*,b*_*m*_] with $b_{m} = v - t\left( {b_{m} } \right)\left( {g + h} \right) < \, v - t\left( {b_{m} } \right)g$ and $s_{m} \le h + g \le v - g < \, v - t\left( {b_{m + 1} } \right)g$. Hence, expected market clearing prices are lower than for *h* = 0 in this case as well.□

#### *Remark*

The condition $h \le v - 2g$ allows for capturing the relevant case that introducing the externality reduces trading volume. Suppose, for instance, that $h = v - 2g$. Then, the minimal willingness to pay of a buyer is $v - g - h = g$ and the maximal willingness to let by a seller is $g + v - 2g = v - g$. As $g < v/2$ this implies a positive probability that not all traders on the shorter market side actually trade at the market clearing price. If *h* becomes too large, the following effect becomes relevant: Suppose buyer and seller types are such that only 1 unit is traded (i.e., *b*_1_ > *s*_1_ and *b*_2_ < *s*_2_) and suppose that *s*_1_ > *b*_2_ and *s*_2_ > *b*_1_ (i.e., market clearing prices are in [*s*_1_,*b*_1_]). Observe that the probability for these inequalities to hold simultaneously conditional on trade taking place converges to 1 as *h* becomes large. As the type *t*(*b*_1_) of the buyer with willingness to pay *b*_1_ is the lowest of *n* draws from *G*(*t*) and the type *t*(*s*_1_) of the seller with costs *s*_1_ is the lowest of *m* draws from $G\left( t \right),t\left( {b_{1} } \right) < t\left( {s_{1} } \right)$ for *n* > *m*. As *h* increases, the lower bound of market clearing prices increases proportional to *t*(*s*_1_) and the upper bound of market clearing prices decreases proportional to *t*(*b*_1_). As a result, the lower bound increases more steeply than the upper bound decreases and the expected market clearing price raises in *h*. The intuition is simple: If *h* is so large that only one pair of buyer and seller trade at most, the market clearing price is no longer determined by the willingness to pay (or to let) of other buyers and sellers but by the willingness to pay and the costs of the two trading parties. However, if the buyer has a lower aversion against generating the externality (and thus a higher willingness to pay despite a given externality), prices increase in the size of the externality. Likewise, prices decrease if there are more sellers than buyers in this case.

### Appendix 2: Additional descriptive statistics

In Tables 5, 6 and 7, we show how many subjects concluded how many trades in each of the six treatments.

**Table 5:** Trading behavior—SYMM and SYMM_EXT

Number of trades	SYMM		SYMM_EXT	
	Number of subjects	Percentage	Number of subjects	Percentage
0	0	0.00	11	13.75
1	0	0.00	0	0.00
2	0	0.00	1	1.25
3	0	0.00	2	2.50
4	0	0.00	4	5.00
5	0	0.00	6	7.50
6	0	0.00	8	10.00
7	0	0.00	6	7.50
8	2	2.50	10	12.50
9	14	17.50	6	7.50
10	64	80.00	26	32.50
Total	80	100.00	80	100.00

**Table 6:** Trading behavior—6SELLERS and 6SELLERS_EXT

Number of trades	6SELLERS		6SELLERS_EXT	
	Number of subjects	Percentage	Number of subjects	Percentage
0	1	0.83	13	10.83
1	0	0.00	4	3.33
2	1	0.83	3	2.50
3	5	4.17	2	1.67
4	4	3.33	8	6.67
5	10	8.33	3	2.50
6	14	11.67	8	6.67
7	10	8.33	8	6.67
8	12	10.00	16	13.33
9	11	9.17	15	12.50
10	52	43.33	40	33.33
Total	120	100.00	120	100.00

**Table 7:** Trading behavior—6BUYERS and 6BUYERS_EXT

Number of trades	6BUYERS		6BUYERS_EXT	
	Number of subjects	Percentage	Number of subjects	Percentage
0	1	0.83	10	8.33
1	1	0.83	6	5.00
2	2	1.67	3	2.50
3	4	3.33	1	0.83
4	9	7.50	5	4.17
5	7	5.83	3	2.50
6	5	4.17	3	2.50
7	11	9.17	11	9.17
8	12	10.00	9	7.50
9	13	10.83	17	14.17
10	55	45.83	52	43.33
Total	120	100.00	120	100.00

## Footnotes

1 Note that Falk and Szech (2013) compare their mouse paradigm with a market where participants can trade vouchers for a university gift shop. In the latter treatment, they do not observe falling prices, and hence interpret the decline in prices in the mouse paradigm as a decay in morals. However, the mouse paradigm differs from the treatment with vouchers in three aspects, thus making causal inference difficult. The first change is that the mouse paradigm has an externality, while the voucher treatment has not. Second, the type of good traded on the market differs (mouse vs. voucher), and third, the traded good has a fixed, and exogenously given, nominal monetary value in one case (the voucher), but cannot unambiguously be monetized in the other case (the life of a mouse). For an elaborate discussion see Breyer and Weimann (2015).

2 In this previous paper (Kirchler et al. 2016) we address the question which kind of interventions can reduce the willingness to trade when trading causes negative externalities. There is a single treatment from Kirchler et al. (2016) that we also use here (the one that is called 6SELLERS_EXT below). We conducted additional experimental sessions for the current paper, and all other five treatments introduced below are novel and address a fundamentally different question from Kirchler et al. (2016).

3 The questions on the assessment of the moral dimension read as follows: “On a scale from 0 (very immoral) to 6 (very moral): How moral do you consider people who have traded in this experiment? On a scale from 0 (very immoral) to 6 (very moral): How moral do you consider people who have NOT traded in this experiment?” The average scores were 3.05 for the first question and 4.62 for the second question (Wilcoxon signed ranks test, *p* < 0.001, *N* = 255 respondents). For details see Kirchler et al. (2016).

4 Instructions for the side experiments are available upon request.

5 The 9 participants left in 4 out of 32 sessions. We do not find a significant difference in trading volume or prices between sessions where at least one participant left and those sessions where all participants continued with the experiment.

6 See also the additional descriptive statistics provided in Appendix 2, where we show that subjects on the short market side who refuse to trade reduce the trading volume significantly.

7 In the Appendix 2, we show in Tables 5, 6 and 7 how many subjects concluded how many trades in each of our six treatments.

8 It is noteworthy, that the prices in the case of six sellers are a mirror image of the prices with six buyers, with no significant differences. For instance, sellers earn on average 7.34 Euro in 6SELLERS_EXT, which is not significantly different from buyers’ mean earnings of 6.44 Euro in 6BUYERS_EXT (*p* = 0.356; Mann–Whitney *U* test, *N* = 24). The same pattern holds also for markets without externalities where sellers earn on average 5.64 Euro in 6SELLERS, and buyers earn 5.13 Euro in 6BUYERS (*p* = 0.525; Mann–Whitney *U* test, *N* = 24).

9 Wilcoxon signed ranks tests of mean prices per market: *p* < 0.001 for SYMM versus 6SELLERS (*N* = 20); *p* < 0.001 for SYMM versus 6BUYERS (*N* = 20); *p* < 0.001 for 6BUYERS versus 6SELLERS (*N* = 24); *p* = 0.007 for SYMM_EXT versus 6SELLERS_EXT (*N* = 20); *p* = 0.002 for SYMM_EXT versus 6BUYERS_EXT (*N* = 20); *p* < 0.001 for 6SELLERS_EXT versus 6BUYERS_EXT (*N* = 24).

10 See also Appendix 2, where we analyze the relative impact of subjects refusing to trade on the longer or shorter market side, respectively, on trading volume and prices. Subjects refusing to trade on the longer market side can drive prices closer to the equal split, i.e., to “fairer” market prices.

11 Using an Ordered probit regression-model for the number of trades per period does *not* change the results. Results for the alternative model are available upon request.

12 Personal background data was not recorded in one session, i.e. for two markets, unfortunately. For this reason, we have only 300 observations instead of 320 in the analysis of Table 4.
